# Large-Scale Fabrication of Nanostructure on Bio-Metallic Substrate for Surface Enhanced Raman and Fluorescence Scattering

**DOI:** 10.3390/nano9070916

**Published:** 2019-06-26

**Authors:** Libin Lu, Jiaru Zhang, Lishi Jiao, Yingchun Guan

**Affiliations:** 1 School of Mechanical Engineering & Automation, Beihang University, Beijing 100191, China; 2 School of Mechanical and Aerospace, Nanyang Technological University, Singapore 639798, Singapore; 3 National Engineering Laboratory of Additive Manufacturing for Large Metallic Components, Beihang University, Beijing 100191, China; 4 Hefei Innovation Research Institute of Beihang University, Xinzhan Hi-tech District, Hefei 230013, China

**Keywords:** hierarchical LIPSS, surface-enhanced Raman scattering, surface-enhanced fluorescence, bio-metallic substrate, crystal violet

## Abstract

The integration of surface-enhanced Raman scattering (SERS) and surface-enhanced fluorescence (SEF) has attracted increasing interest and is highly probable to improve the sensitivity and reproducibility of spectroscopic investigations in biomedical fields. In this work, dual-mode SERS and SEF hierarchical structures have been developed on a single bio-metallic substrate. The hierarchical structure was composed of micro-grooves, nano-particles, and nano-ripples. The crystal violet was selected as reporter molecule and both the intensity of Raman and fluorescence signals were enhanced because of the dual-mode SERS−SEF phenomena with enhancement factors (EFs) of 7.85 × 10^5^ and 14.32, respectively. The Raman and fluorescence signals also exhibited good uniformity with the relative standard deviation value of 2.46% and 5.15%, respectively. Moreover, the substrate exhibited high sensitivity with the limits of detection (LOD) as low as 1 × 10^−11^ mol/L using Raman spectroscopy and 1 × 10^−10^ mol/L by fluorescence spectroscopy. The combined effect of surface plasmon resonance and “hot spots” induced by the hierarchical laser induced periodical surface structures (LIPSS) was mainly contributed to the enhancement of Raman and fluorescence signal. We propose that the integration of SERS and SEF in a single bio-metallic substrate is promising to improve the sensitivity and reproducibility of detection in biomedical investigations.

## 1. Introduction

Recently, surface-enhanced Raman scattering (SERS) has received increased research interest, as it can allow for high sensitivity and selectivity in biochemical detection such as detection of carbaryl pesticide residues, exploring the metabolism of bacterial cells and so on [1,2,3,4]. The enhancement factors (EFs) of SERS is up to 10^14^, which makes it possible to detect a single molecule [5]. However, the long measurement time together with the undesirable image resolution impede its practical applications in biomedical fields. Surface enhanced fluorescence (SEF) is another powerful spectroscopic method in the selective detection of bioanalytes. The intense electric field near the metallic tip can strongly enhance the stimulating energy for the fluorescence molecular. Although the SERS and SEF have promising potential in detecting biomedical signals, it is certainly challenging to study the two spectroscopic approaches together [6,7,8].

Recently, the development of a multifunctional substrate that integrates various individual functions into a single system attracts strong attention in nanotechnology fields [9,10]. It is reported that the sensitivity of biomedical spectroscopic is improved by the combination of the superior advantages and synergistic effects of SERS and SEF. Kamalieva et al. [11] developed a composite structure based on silver nanoparticles and a thin protective silicon film. In Kamalieva et al.’s research, the Raman scattering and fluorescence signal of cyanine-dye emitters on formed composite structure were enhanced by factors of 10 and 40, respectively. However, the obtained values of both fluorescence and Raman scattering enhancement were too low compared to the previously reported results. Cao et al. [12] integrated metal-enhanced fluorescence and Raman functions in a gold and gelatin core-shell nanostructures. The enhancement factors of Raman and fluorescence signals were found to be 3.1 × 10^4^ and 4-fold, respectively. Furthermore, Cyrankiewicz et al. [13] studied the enhancement properties of silver nanoparticles in SERS and metal-enhanced fluorescence (MEF). They found that the agglomeration of nanoparticles (so-called “hot spots”) was a prerequisite for efficient SERS. The maximal enhancement factor of Raman and fluorescence was 10^6^ and 9-fold, respectively. In Change et al.’s study [14], silver nanoparticles demonstrate both SERS and SEF properties. They have found that metallic particles’ aggregation status together with the interval between metal structure and emitters strongly influence the enhancement ratio.

Although many studies have been carried out to integrate SERS and SEF in a single platform, almost all the multi-functional platform are metallic nanoparticles in solution. The main drawback for this platform lies in the aggregation of metallic nanoparticles which can result in a poor reproducibility of the prepared samples. Moreover, the fabrication process of nanoparticles is very complicated and their biocompatibility needs to be further improved. That might be the limitation for the practical applications of SERS and SEF systems in biomedical investigations.

In the current work, we fabricated a hierarchical LIPSS structure on Ti6Al4V substrate, which can provide dual-mode enhanced spectroscopic properties by SEF and SERS. The proposed hierarchical LIPSS was composed of micro-grooves, nano-particles, and nano-ripples. Crystal violet (CV) was chosen as species reporter molecule to indicate Raman and fluorescent signal. The combined effect from surface plasmon resonance and “hot spots” on enhancement factors of Raman and fluorescence signals is studied. The results shows that the proposed SERS and SEF dual-mode bio-metallic substrate could have great promise for ultra-sensitive detection in the biomedical investigations, such as biological sensor, photonics, bioimaging, and so on.

## 2. Materials and Methods

### 2.1. Sample Preparation

The experiments were carried out on biomedical grade 5 Ti6Al4V alloy substrate with a thickness of 1 mm and an area of 10 × 10 mm^2^. The Ti6Al4V is one of the most common biomaterials and is widely used in the biomechanical area. Before the laser processing, the Ti alloy samples were polished with a 400 to 2000 grit sequence of SiC papers. Then, the polished samples were cleaned in ultrasonic bath for 5 min in alcohol. Large-area hierarchical structures were fabricated by Yb:KGW solid-state laser diode (Pharos, Light Conversion) with a maximum power of 20 W and pulse duration of 230 fs in ambient air. The central wavelength of laser was 1030 nm and the beam diameter was 35 μm.

### 2.2. Surface Characterization

Surface morphology of the irradiated areas were analyzed by a scanning electron microscope (SEM, Quanta 450 FEG, FEI, Hillsboro, OR, USA). Atomic force microscopy (AFM, ICON, Bruker, Madison, WI, USA) and 3D laser scanning confocal microscope (VK100, Keyence, Osaka, Japan) were utilized to measure the topography of the hierarchical structure.

### 2.3. Reflectance and Absorption Spectroscopy

The reflectance and absorption spectrum of the untreated surface and prepared hierarchical LIPSS were recorded by a UV-vis-NIR (Ultraviolet-Visible-Near Infrared) spectrophotometer (UV3600, Shimadzu Scientific Instruments, Kyoto, Japan) with an integrating sphere (MPC-3100) in the wavelength range from 250 to 700 nm.

### 2.4. Raman and Fluorescence Spectroscopy

Crystal Violet (CV) (purity 98%) was purchased from Sigma-Aldrich (Saint Louis, MO, USA). The diluted solutions with various concentrations were prepared in ethanol (99.8% HPLC (High Performance Liquid Chromatography) grade). Droplets of CV solution was placed onto the prepared surface and dried in air. Raman spectra were measured using a Raman microscope (HR800, Jobin Yvon, Paris, France). A He-Gd laser operating at wavelength of 325 nm was applied for the Raman excitation. An 1800 g/mm diffraction grating is used to disperse the Raman spectra onto the CCD (Charge-coupled Device). The excitation laser was focused by an objective lens with a numerical aperture of 50, which was also used to collect scattering light (Raman signal) in back-reflection. Fluorescence spectrum with an excitation wavelength of 580 nm was recorded from a fluorescence spectrophotometer (F-7000, Hitachi, Tokyo, Japan). For steady-state measurements, the scan speed was set to be 1200 nm/min. The excitation and emission slit width of the fluorescence spectrophotometer were both set to be 5 nm. Unless specially emphasized otherwise, the above conditions were applied for all Raman and fluorescence measurements.

## 3. Results and Discussion

### 3.1. Topography of the Hierarchical LIPSS

**Figure 1:**
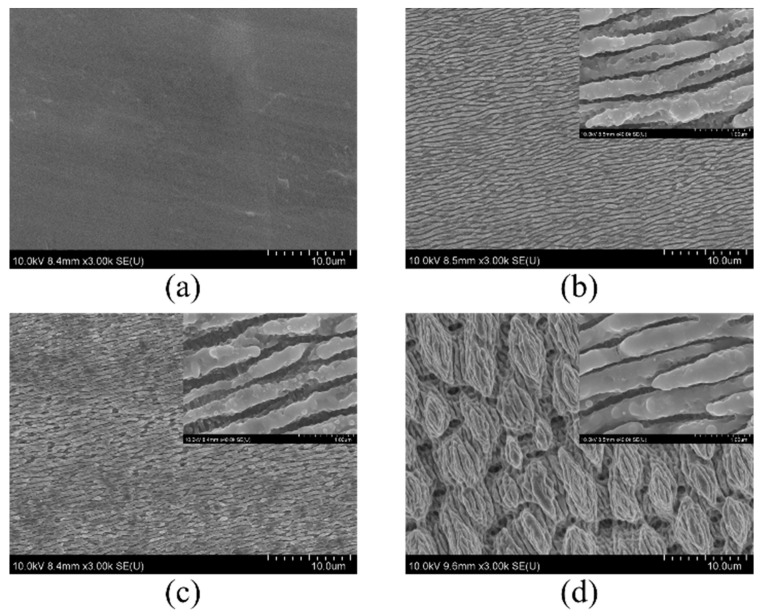
Morphological evolution of Ti6Al4V surface after irradiation with progressively laser scan times: (**a**) untreated; (**b**) 5 cycles; (**c**) 20 cycles; (**d**) 30 cycles.

Figure 1 shows the evolution of hierarchical LIPSS at various laser scanning cycles. Figure 1b shows the LIPSS scanned by five cycles with an average laser fluence of 0.12 J/cm^2^, which was slightly higher than the material damage threshold (~0.1 J/cm^2^) [15]. The formation of LIPSS on the substrate was mainly attributed to the interference between incident laser and the laser induced surface plasmon wave [16]. During the interference process, the laser intensity was redistributed into a periodic pattern on the air solid interface. The materials around laser intensity peak that is above the damage threshold could be removed and LIPSS were therefore formed [17,18,19]. Hierarchical LIPSS consisted of micro-grooved surfaces covered by nano-particles and nano-ripples were obtained at scanning cycles of 20, as shown in Figure 1c. With further increasing the scanning cycles to 30, however, the LIPSS was destroyed due to the excessive laser energy input. The inset of Figure 1 shows that LIPSS structures were covered by random nanoparticles, which were believed to cause high EFs due to the creation of “hot spots” [20]. It is believed that the nanoparticles are generated by laser caused fluid fragmentation [21]. During the femtosecond laser-surface interaction, the laser energy was rapidly deposited onto the substrate, causing lattice vibration and temperature rise in short time. The energetic melting fluid with high pressure was thereby created. Then the fluid expanded into the air, the mechanical bonds were broken by the strain associated with the fast expansion of melting, which resulted in fluid fragmentation [22,23]. The particle size was measured manually using an open source image processing software ImageJ (NIH, Bethesda, MD, USA), where a total of ∼100 nano-particles were measured to obtain an average dimension. As shown in Figure 1, three LIPSS substrates were fabricated at laser scanning cycles of 5, 20, and 30. The nano-particles density first increased from 6.5 × 106/mm^2^ to 9.3 × 106/mm^2^, then decreased to 2.9 × 106/mm^2^ and the average diameter of nano-particles was 41 nm, 48 nm, and 130 nm, respectively. Previous studies show that nano-particles with a diameter of 50 nm produced the maximum SERS enhancement [24]. In order to further investigate the LIPSS’s enhancement effect in Raman and fluorescence, in the following studies, the laser scanning cyclesof 20 that can produce the 48 nm nanoparticle is selected as the optimal experimental condition to prepare the substrate.

**Figure 2:**
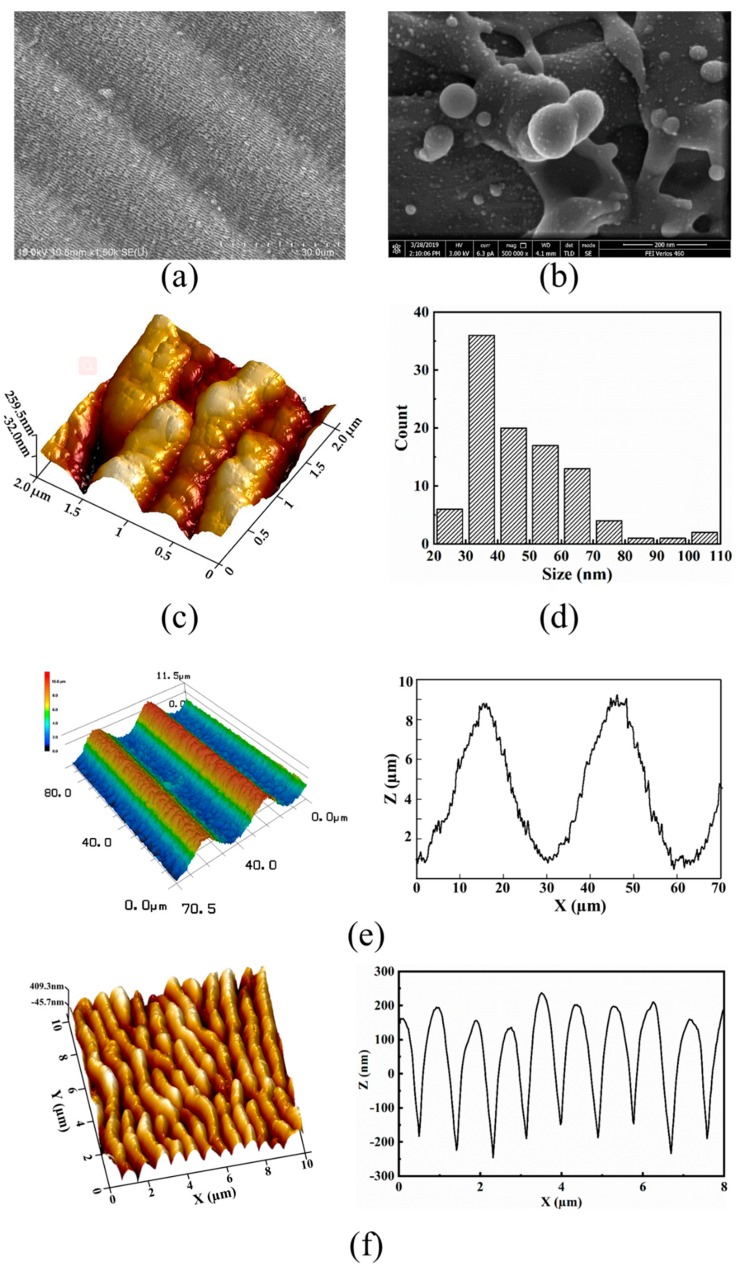
Micrograph of hierarchical laser induced periodical surface structures (LIPSS): (**a**) 45° view of scanning electron microscope (SEM) image; (**b**) magnification of nano-particles; (**c**) magnification of LIPSS; (**d**) the size distributions of nanoparticles; (**e**) 3D surface topography (left), height profiles of hierarchical LIPSS (right); (**f**) Atomic force microscopy image of the LIPSS; height profiles of nano-ripples (right).

The 45° view of hierarchical LIPSS were shown in Figure 2a and magnification of nano-particles and LIPSS were shown in Figure 2b,c. Nano-particle aggregation was clearly seen in Figure 2b. This aggregation can concentrate the incident electromagnetic field and effectively amplify the near field between and around the nanoparticles, which is believed to be associated with the “hot spots” [25]. It was found that the size distribution of nano-particles varied from 20 nm to 110 nm. The average periodicity and depth of the micro-grooves was 30 µm and 8 µm, respectively, as illustrated in Figure 2e. In Figure 2f, the spatial period of nano-ripples was found to be 890 nm, which was less than the laser wavelength of 1030 nm. This could be attributed to the increment of the real part of the material refractive index due to the higher surface roughness during the laser processing [26,27].

### 3.2. Optical Properties

**Figure 3:**
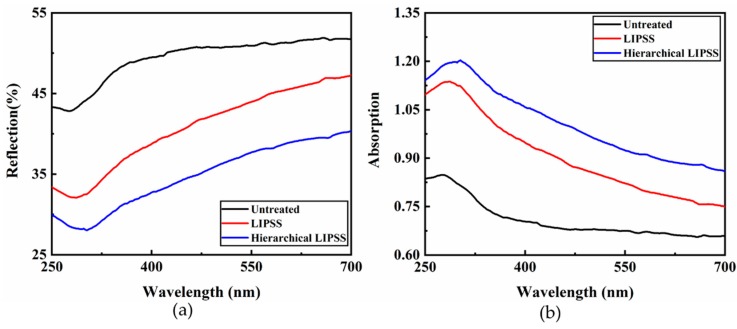
The reflection (**a**) and absorption (**b**) spectra of untreated, LIPSS, and hierarchical LIPSS substrate.

To investigate the far-field optical properties of the fabricated structures and select the optimal wavelength to excite the Raman signal, the optical absorption and reflection properties of hierarchical LIPSS, LIPSS, and untreated substrate were measured in the wavelength range from 250 to 700 nm, as presented in Figure 3. Compared to the untreated substrate, the reflection of hierarchical LIPSS and LIPSS substrates decreased by more than 15% and 10%, respectively. This reflection decrease was attributed to the localized surface plasmon resonance (LSPR) induced change in optical absorptive properties [28]. Because of the efficient LSPR excitation resulting from the micro-grooves, the hierarchical LIPSS substrate showed lower reflection values than those of LIPSS. As indicated in Figure 3b, a red shift of the spectrum peak can be seen from LIPSS and hierarchical LIPSS. This phenomenon is because of the interaction among the LIPSS. Moreover, the hierarchical LIPSS and LIPSS substrate showed broadband light absorption, which was attributed to the broad size distribution of the nanostructures that can cause surface plasmon resonant at various wavelength [29].

In Figure 3, the maximum absorption wavelength of untreated, LIPSS, and hierarchical LIPSS substrate are 278 nm, 287 nm, and 305 nm, respectively. It is generally known that the common laser excitation wavelengths used in the Raman microscope were 325 nm, 532 nm, 633 nm, and 785 nm, respectively. According to the absorption spectra of the substrates, the light absorption at 325 nm wavelength appeared to be higher than that at other common wavelengths, which was beneficial to the surface plasmon resonance. Therefore, 325 nm excitation was chosen for the Raman test.

### 3.3. SERS Characteristics

**Figure 4:**
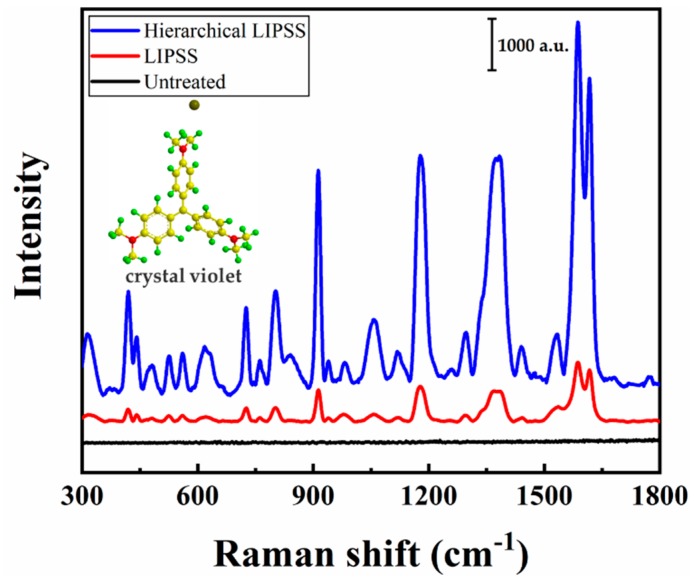
Raman spectra obtained from untreated, LIPSS, and hierarchical LIPSS substrates, respectively.

The CV solution was used to investigate the sensitivity and reproducibility of SERS signal. As shown in Figure 4, the spectra in the range from 300 to 1800 cm^−1^ were recorded with 20 µL of CV solution at a concentration of 10^−3^ mol/L dropped onto the untreated, LIPSS, and hierarchical LIPSS substrate. It can be seen that the typical Raman peaks of CV located at 802, 913, 1175, 1229, 1539, 1583, and 1618 cm^−1^ were clearly observed [30,31]. The results showed that the intensity of the Raman signal was greatly increased with the use of LIPSS and hierarchical LIPSS substrate. Moreover, the hierarchical LIPSS substrate exhibited a much higher Raman intensity than that of LIPSS substrate. This significant enhancement indicated that hierarchical nanostructure played a very important role in the generation of electromagnetic enhancement that induced a significant enhancement of SERS signals from the adsorbed CV molecules.

The enhancement factors (EFs) were used to quantify the Raman enhancement of different substrates and were estimated by the following formula [32,33]:

(1) $$EF_{SERS}=\frac{I_{SERS}}{I_{Raman}}\times \frac{N_{Raman}}{N_{SERS}}$$

where *I_SERS_* and *I_Raman_* are the intensities of the selected Raman peaks on SERS and untreated substrate, *N_SERS_* and *N_Raman_* are the number of CV molecules adsorbed on SERS and untreated substrate. The average number of adsorbed CV molecules (*N*) in laser illumination volume of testing areas was estimated by the following formula

(2) N=cV

where *c* and *V* are the concentration of CV molecules and laser illumination volume, respectively. It is assumed that the CV molecules are distributed homogenously on different substrates once the CV solution was dropped and dried [34,35,36]. Therefore, the EFs can be estimated by the following formula [37]:

(3) $$EF_{SERS}=\frac{I_{SERS}}{I_{Raman}}\times \frac{C_{Raman}}{C_{SERS}}$$

where *C_SERS_* and *C_Raman_* are the concentration of CV solution used for SERS and untreated substrate, respectively. In this work, the intensity of peaks located at 913, 1175, 1229, 1539, 1587, and 1618 cm^−1^ were used to estimate the EFs. As listed in Table 1, the calculated EFs of CV on LIPSS and hierarchical LIPSS substrate are up to 3.59 × 10^4^ and 7.85 × 10^5^, respectively.

The excitation of surface plasmon resonance (SPR) with a strong field enhancement, known as electromagnetic mechanism, is the main contribution to SERS enhancement [38]. It is known that the SPR can occur in two different forms: the localized SPR (LSPR) and the propagating surface plasmon polariton (SPP) [39]. In this work, the LSPR occurs when the dimension of the nanoparticles (average size 48 nm) was much less than the incident light wavelength (325 nm). In this range, the electron displacement against the atomic cores led to collective but non-propagating surface electrons oscillations in the nanostructure [40,41]. Strong local electric fields induced from the LSPR is the reason for the enhanced signal. Moreover, the sparse and randomly distributed nano-particles (known as “hot spots”) could also cause small areas of greatly enhanced electromagnetic field [42]. Therefore, the Raman signal was enhanced on the hierarchical LIPSS and LIPSS substrates. Moreover, when hierarchical LIPSS was illuminated by incident light, the surface plasmon mode generated in nano-particles could also propagate along the micro-grooves, which produced more “hot spots” and the local electric fields was further enhanced in hierarchical LIPSS [43]. Furthermore, the hierarchical LIPSS could provide larger surface areas to deposit more “hot spots” [44]. Thus, the stronger localized electric fields in the hierarchical LIPSS produced higher Raman enhancement than LIPSS. The SPP induced by the nano-ripples and micro-grooves was also responsible for the stronger Raman signal. The wave-vector of SPP can be estimated by the following formula:

(4) $$k_{spp}=\frac{2\pi }{\lambda }\sqrt{\frac{\varepsilon_{m}\varepsilon_{e}}{\varepsilon_{m}+\varepsilon_{e}}}$$

where *λ*, *ε_m_* and *ε_e_* are the wavelength of the incident light, the permittivity of the substrate and CV, respectively.

The wavelength of SPP can be estimated by employing the following formula:

(5) $$\lambda_{spp}=\frac{2\pi }{k_{spp}}=\lambda \sqrt{\frac{\varepsilon_{m}+\varepsilon_{e}}{\varepsilon_{m}\varepsilon_{e}}}$$

In the current work, the *λ*, *ε_m_*_,_ and *ε_e_* is 325 nm, 114 [45], and 5 [46], respectively. According to Equation (4), the λ_spp_ is 147.50 nm. The period of nano-ripples and microgrooves is 890 nm and 31 µm, respectively, which is the 6.03 and 210.17 times of the SPP wavelength. Considering the measuring errors, the grating period was 6 and 210 multiples of the SPP wavelength, respectively, which could effectively excite the SPP [47].

**Figure 5:**
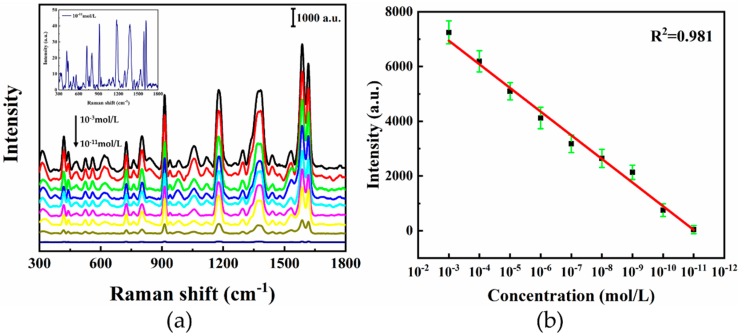
(**a**) SERS spectra of CV with the concentration decreased from 10^−3^ to 10^−11^ mol/L. Inset shows the detailed view of SERS spectra of CV at 10^−11^ mol/L; (**b**) plot of Raman intensities for CV at 1583 cm^−1^ with different concentrations.

The sensitivity of the SERS substrate was studied with different CV concentrations ranging from 10^−11^ mol/L to 10^−3^ mol/L dropped onto the hierarchical LIPSS substrate and their Raman spectra under 325 nm excitations were recorded in Figure 5a. The intensity of Raman signal decreased with decreasing CV concentration. The inset picture in Figure 5a showed that the spectra peaks of CV with a concentration of 10^−11^ mol/L. The plot of Raman intensities for CV at 1583 cm^−1^ showed a good linear correlation between the SERS intensity and the CV concentration ranging from 10^−3^ to 10^−11^ mol/L. As illustrated in Figure 5b, the linear equation was y = 9537.74 + 863.95x (x represented the logarithm of concentration, y represented the Raman intensity at 1587 cm^−1^). As shown in Figure 5a, the limit of detection (LOD) of CV was about 1 × 10^−11^ mol/L, which was comparable to the previous results (from 10^−14^ mol/L to 10^−6^ mol/L) [48,49]. Therefore, the SERS substrate shows good sensitivity for detecting CV molecules at low concentration.

**Figure 6:**
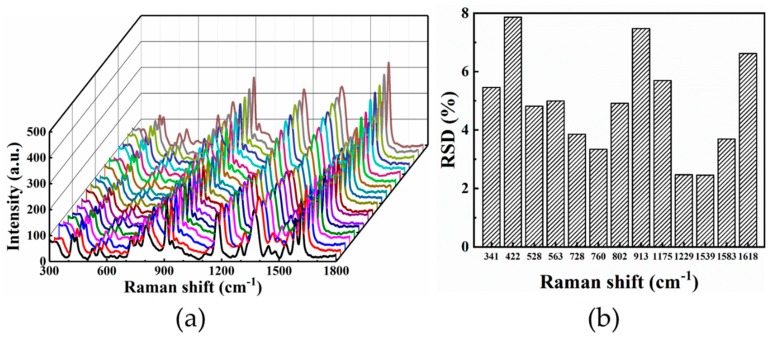
(**a**) SERS spectra of CV with the concentration of 10^−10^ mol/L absorbed on hierarchical LIPSS collected from 20 locations from the substrate; (**b**) RSD(relative standard deviation) value of the major Raman peaks of CV spectrum.

In order to study the reproducibility for SERS testing, 20 different locations on the substrate were randomly selected to collect the SERS signal at CV concentration of 10^−10^ mol/L, as illustrated in Figure 6a. The major band of CV was used to evaluate the reproducibility of the Raman signal intensity, as shown in Figure 6b. A maximum RSD (relative standard deviation) value of 7.87% for Raman intensities of the 422 cm^−1^ was obtained. Meanwhile, the SERS intensities at 1539 cm^−1^ showed the lowest RSD value of 2.46%. Hence, the hierarchical LIPSS substrate could work as a substrate with high sensitivity as well as good reproducibility in detecting CV molecules.

### 3.4. SEF Characteristics

**Figure 7:**
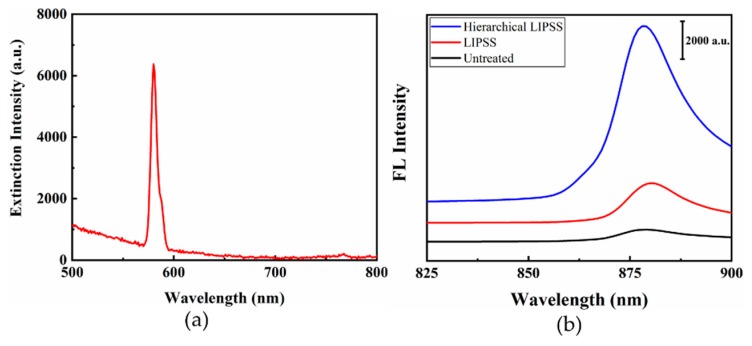
(**a**) Fluorescence extinction spectra of CV; (**b**) Fluorescence spectra of CV on the untreated, LIPSS, and hierarchical LIPSS, with excitation wavelength at 580 nm.

The CV molecules were also used to investigate the surface enhanced fluorescence (SEF) effects on the different substrates by detecting the intensity of fluorescence spectrum. The fluorescence extinction spectra of CV adsorbed on hierarchical LIPSS substrate was shown in Figure 7a. At the peak of 580 nm, the CV on the hierarchical LIPSS exhibited obvious plasmon resonance bands due to the electron oscillations [50]. According to the extinction spectra, the 580 nm wavelength was used for extinction emission spectrum of CV. As shown in Figure 7b, 20 µL of CV solution at a concentration of 10^−3^ mol/L was dropped onto the different substrates and the fluorescence spectra ranging from 825 to 900 nm were recorded. It is found that both LIPSS and hierarchical LIPSS substrates could enhance the fluorescence of CV at 880 nm. It should be noted that the fluorescence on the hierarchical LIPSS substrate showed larger enhancement than that from LIPSS substrate.

By comparing the peak at 880 nm, the EF is found to be around 3.25 and 14.32 for LIPSS and hierarchical LIPSS based on the following equation:

(6) $$E_{SEF}=\frac{I_{SEF}-I_{background}}{I_{reference}-I_{background}}$$

where *I_SEF_* is the fluorescence intensity of CV from LIPSS or hierarchical LIPSS, *I_reference_* is the fluorescence intensity from CV, and *I_background_* is the background spectra intensity.

The LSPR from the collective electrons oscillation of the nanostructures was considered to be the mainly reason for the SEF enhancement [51]. As illustrated in Section 3.3, the LSPR and “hot spots“ could induce extremely high local electric fields, leading to the an enhanced excitation rates and efficiency [52]. Then, the fluorescence emission coupled surface plasmon caused the incensement of radiative decay rate, leading to the fluorescence signal enhancement [53]. The increased fluorophores radiative decay rate and enhanced local field would increase both the quantum yield rates and the excitation efficiency, resulting in the enhancement of the fluorescence emission of CV near LIPSS. Moreover, the hierarchical LIPSS could enlarge the surface area with more complicated morphology, leading to stronger coupling effect with fluorophores and localized fields. Therefore, the hierarchical LIPSS substrate produced higher fluorescence enhancement than LIPSS.

**Figure 8:**
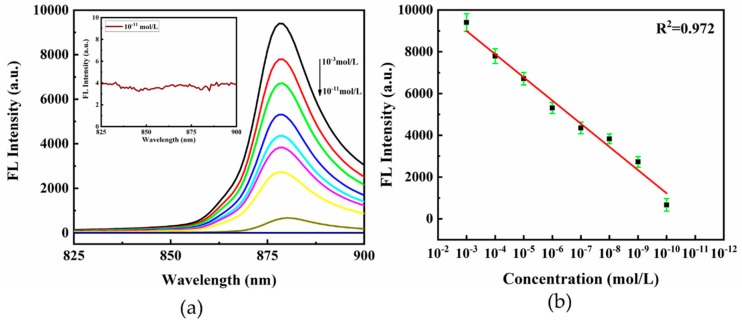
(**a**) Fluorescence spectra of CV at different concentrations (from 10^−3^ to 10^−11^ mol/L). Insets show the detailed view of fluorescence spectra of CV at 10^−11^ mol/L; (**b**) linear correlation of fluorescence intensities at 880 nm.

Figure 8a shows the SEF spectra of CV with the concentration from 10^−3^ to 10^−11^ mol/L using the hierarchical LIPSS substrate. The fluorescence signal of CV with the concentration of 10^−11^ mol/L was barely observed, which indicated that the LOD was about 1 × 10^−10^ mol/L, as shown in the inset of Figure 8. The plot of fluorescence intensities for CV also showed a well linear correlation with CV concentration ranging from 10^−3^ mol/L to 10^−10^ mol/L, as shown in Figure 8b. The linear equation was y = 12329.96 + 1110.56x (x was the logarithm of CV concentration; y was the fluorescence intensity; the correlation coefficient R^2^ was 0.972).

**Figure 9:**
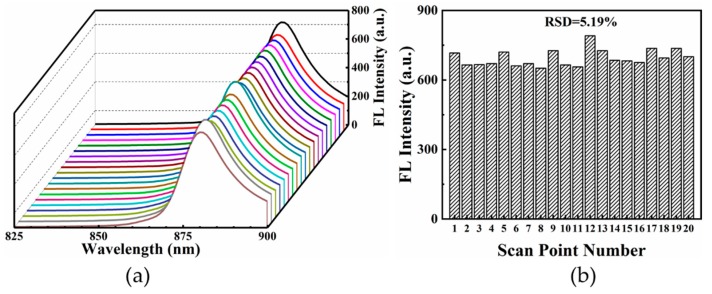
(**a**) Fluorescence spectra of 10^−10^ mol/L CV absorbed on hierarchical LIPSS collected from twenty randomly selected acquisition points from substrates; (**b**) the RSD of the integrated fluorescence intensity of CV at 880 cm^−1^.

To test the reproducibility of SEF substrates, fluorescence spectra of CV (1 × 10^−10^ mol/L) were collected from twenty different locations on the hierarchical LIPSS substrate, as illustrated in Figure 9a. In column graphs of Figure 9b, the relative RSD estimated by the fluorescence intensities of 880 nm was 5.19%. These results indicated the hierarchical LIPSS substrate also had favorable sensitivity and reproducibility for detecting CV fluorescence.

## 4. Conclusions

In this work, we have studied the hierarchical LIPSS structure fabricated by femtosecond laser on Ti6Al4V substrate with the purpose to provide a proof for integrating both SERS and SEF’s enhancement capabilities into a single substrate, in which CV was employed as Raman and fluorescent probes. The main conclusions are listed as follows:

1. The hierarchical LIPSS structure consisting of micro-grooves, nano-ripples, and nano-particles with strong SPR was produced by one-step femtosecond laser processing, which may open up new possibilities in both SERS and SEF.
2. Due to the combination effect of SPR and “hot spots”, the hierarchical LIPSS substrate exhibits an ultra-sensitive detectability, which shows an EFs of 7.85 × 10^5^ for CV and LOD of 10^−11^ mol/L.
3. The intensity of the CV fluorescence on the hierarchical LIPSS substrate was enhanced by about 14 times with the LOD of 1 × 10^−10^ mol/L, which is attributed to the LSPR and “hot spots” from the hierarchical LIPSS.

## Figures and Tables

**Table 1:** Peak position value of the surface-enhanced Raman scattering (SERS) spectra and calculated enhancement factors (EFs) of Crystal Violet (CV) adsorbed on LIPSS and hierarchical LIPSS substrate.

Peak (cm^−1^)	913	1175	1229	1539	1587	1618
LIPSS	8.83 × 10^3^	1.99 × 10^4^	2.15 × 10^3^	8.29 × 10^3^	3.59 × 10^4^	2.16 × 10^4^
Hierarchical LIPSS	2.82 × 10^4^	6.27 × 10^4^	7.85 × 10^5^	7.28 × 10^4^	2.32 × 10^5^	1.36 × 10^5^
